# The Role of 5-ALA in Low-Grade Gliomas and the Influence of Antiepileptic Drugs on Intraoperative Fluorescence

**DOI:** 10.3389/fonc.2019.00423

**Published:** 2019-05-22

**Authors:** Sergey A. Goryaynov, Georg Widhalm, Maria F. Goldberg, Danil Chelushkin, Aldo Spallone, Kosta A. Chernyshov, Marina Ryzhova, Galina Pavlova, Alexander Revischin, Ludmila Shishkina, Vadim Jukov, Tatyana Savelieva, Loschenov Victor, Alexander Potapov

**Affiliations:** ^1^N. N. Burdenko Scientific Research Neurosurgery Institute, Moscow, Russia; ^2^Department of Neurosurgery, Medical University of Vienna, Vienna, Austria; ^3^I.M. Sechenov First Moscow State Medical University, Moscow, Russia; ^4^NCL-Institute of Neurological Sciences, Rome, Italy; ^5^Department of Biomedicine, University of Rome Tor Vergata, Rome, Italy; ^6^Institute of Gene Biology, Russian Academy of Science, Moscow, Russia; ^7^Prokhorov General Physics Institute, Russian Academy of Sciences, Moscow, Russia; ^8^National Research Nuclear University, Moscow Engineering Physics Institute, Moscow, Russia

**Keywords:** low-grade glioma, 5-aminolevulinic acid, protoporphyrin IX, fluorescence, antiepileptic drugs

## Abstract

**Objectives:** Intraoperative tumor visualization with 5-aminolevulinic acid (5-ALA) induced protoporphyrin IX (PpIX) fluorescence is widely applied for improved resection of high-grade gliomas. However, visible fluorescence is present only in a minority of low-grade gliomas (LGGs) according to current literature. Nowadays, antiepileptic drugs (AEDs) are frequently administered to LGG patients prior to surgery. A recent *in-vitro* study demonstrated that AEDs result in significant reduction of PpIX synthesis in glioma cells. The aim of this study was thus to investigate the role of 5-ALA fluorescence in LGG surgery and the influence of AEDs on visible fluorescence.

**Patients and Methods:** Patients with resection of a newly diagnosed suspected LGG after 5-ALA (25 mg/kg) administration were initially included. During surgery, the presence of visible fluorescence (none, mild, moderate, or bright) within the tumor and intratumoral fluorescence homogeneity (diffuse or focal) were analyzed. Tissue samples from fluorescing and/or non-fluorescing areas within the tumor and/or the assumed tumor border were collected for histopathological analysis (WHO tumor diagnosis, cell density, and proliferation rate). Only patients with diagnosis of LGG after surgery remained in the final study cohort. In each patient, the potential preoperative intake of AEDs was investigated.

**Results:** Altogether, 27 patients with a histopathologically confirmed LGG (14 diffuse astrocytomas, 6 oligodendrogliomas, 4 pilocytic astrocytomas, 2 gemistocytic astrocytomas, and one desmoplastic infantile ganglioglioma) were finally included. Visible fluorescence was detected in 14 (52%) of 27. In terms of fluorescence homogeneity (*n* = 14), 7 tumors showed diffuse fluorescence, while in 7 gliomas focal fluorescence was noted. Cell density (*p* = 0.03) and proliferation rate (*p* = 0.04) was significantly higher in fluorescence-positive than in fluorescence-negative samples. Furthermore, 15 (56%) of 27 patients were taking AEDs before surgery. Of these, 11 patients (73%) showed no visible fluorescence. In contrast, 10 (83%) of 12 patients without prior AEDs intake showed visible fluorescence. Thus, visible fluorescence was significantly more common in patients without AEDs compared to patients with preoperative AED intake (OR = 0,15 (CI 95% 0.012–1.07), *p* = 0.046).

**Conclusions:** Our study shows a markedly higher rate of visible fluorescence in a series of LGGs compared to current literature. According to our preliminary data, preoperative intake of AEDs seems to reduce the presence of visible fluorescence in such tumors and should thus be taken into account in the clinical setting.

## Introduction

Gliomas are the most common intracranial tumors representing approximately 70% of all primary brain tumors (1, 2). It is well-known that gross-total resection correlates with improved progression-free and overall survival in patients with low-grade gliomas (LGGs) (3–6). Thus, maximum safe resection of LGGs is regarded nowadays as the recommended primary treatment to delay potential malignant transformation (7, 8). However, such maximum safe resection is only achieved in the minority of LGGs due to their infiltrative growth and undefined borders (9).

Surgery using 5-aminolevulinic acid (ALA) induced protoporphyrin IX (PpIX) fluorescence has been introduced to the neurosurgical field for improved intraoperative tumor visualization (10). In the last two decades, such fluorescence-guided resections were especially applied to optimize surgery of high-grade gliomas (HGGs) (11). In this sense, 5-ALA fluorescence-guided surgery results in a significantly higher frequency of complete resections and a prolonged progression-free survival in HGGs (12–17). In the last years, 5-ALA was also increasingly investigated during surgery of radiologically suspected LGGs (3, 7, 11, 13, 18–21). According to the data of these first clinical studies, 5-ALA induced fluorescence is a powerful marker to identify potential regions of malignant transformation (anaplastic foci) during surgery of suspected LGG and thus to avoid histopathological undegrading. However, the majority of pure LGGs cannot be visualized by visible fluorescence according to the current literature (3, 7, 11, 13, 18, 19, 21, 22).

The exact mechanisms of PpIX accumulation and thus the presence or absence of visible 5-ALA induced fluorescence in gliomas are still unclear. A large variety of factors were suspected to influence visible 5-ALA fluorescence such as increased metabolism and up-regulation of porphyrin-producing enzymes (23), reduced iron metabolism within neoplastic cells (24), and reduction of activity of the ferrochelatase enzyme that converts fluorescing PpIX into heme (25). Recently, an *in-vitro* study reported that antiepileptic drugs (AEDs) result in an injury of the mitochondrial membrane and thus lead to inhibition of PpIX synthesis in glioma cells (26). Nowadays, AEDs are frequently administered to patients suffering from LGG prior to surgery. We hypothesize that administration of AEDs might influence the presence of visible fluorescence in LGGs during surgical resection.

The aim of the present study was thus to investigate the role of 5-ALA induced fluorescence in LGG surgery and analyze the influence of AEDs on the presence of visible fluorescence.

## Materials and Methods

### Patient Population

Patients that underwent resection of a newly diagnosed suspected LGG at the Burdenko Neurosurgical Institute after 5-ALA administration between March 2014 and March 2016 were recruited. In our study, altogether 27 patients with a histopathologically confirmed LGG were finally included. Our study cohort included 19 men and eight women with a median age of 33 years (range: 18–66 year). According to our histopathological analysis, 14 diffuse astrocytomas, 6 oligodendrogliomas, 4 pilocytic astrocytomas, 2 gemistocytic astrocytomas, and one desmoplastic infantile ganglioglioma were diagnosed. The application of 5-ALA during surgery was feasible in all 27 patients. In none of our patients, any significant 5-ALA related side effects occurred in our study. Informed consent for the surgical procedure and administration of 5-ALA was obtained from all patients. The study was approved by the local ethics committee of the N. N. Burdenko National Medical Research Center of Neurosurgery (Moscow, Russia).

### Inclusion and Exclusion Criteria

Inclusion criteria for enrolment into this study were age ≥18 years, MRI–suspected LGG, possible gross total resection (GTR) (i.e., >90%) as judged by preoperative surgical estimation, absence of any known history of liver disease or signs of significant hepatic dysfunction and Karnofsky scale ≥70. Exclusion criteria for the administration of 5-ALA were history of photosensitivity, patient, or family history of porphyria, pregnancy, and breast-feeding. Only patients with diagnosis of LGG after surgery remained in the final study cohort.

### Surgical Procedure

Patients were administered orally 25 mg/kg bodyweight 5-ALA (“Alasence” NIOPIK, Moscow, Russia) dissolved in 100 ml of water ~3 h before surgery. Depending on the tumor localization, intraoperative neuromonitoring with sensory/motor evoked potentials, and/or direct stimulation (Viking Select, Nicolet; *n* = 21 patients) as well as awake surgery (*n* = 2 patients) was performed. A modified neurosurgical microscope (Carl Zeiss OPMI Pentero, Germany) equipped with a fluorescent 400 nm UV light module and specific filters were used. Microsurgical tumor removal was performed using primarily standard white light microscopy with assistance of neuronavigation in most cases (*n* = 20 patients). During surgery, the microscope was switched to violet-blue excitation light repeatedly to visualize potential fluorescence.

Fluorescence intensity was visually assessed by a surgeon and determined as “weak” with only a small tumor part showing pink fluorescence with its bulk not fluorescing at all, “moderate” with more than half of the tumor revealing pink fluorescence, and “bright” with the major part of the tumor looking bright red. Spectroscopy-assisted Fluorescence was quantitatively assessed in 12 patients. The level of fluorescence varied from 0 values up to 15 arbitrary units (after data normalization) regarding the intact brain. We specially did not highlight the role of spectroscopy, as it was performed only in some of our patients. Furthermore, also the intratumoral fluorescence homogeneity of each tumor was determined. In this sense, diffuse fluorescence was defined as homogeneous glowing of the whole tumor. In contrast, focal fluorescence was defined as a circumscribed area of fluorescence within an otherwise non-fluorescing tumor. In the course of surgery, tissue samples from fluorescing and/or non-fluorescing areas within the tumor and/or the assumed tumor border were subsequently collected for histopathological analysis. To avoid potential phototoxicity related to 5-ALA, all patients were protected from strong light sources for at least 24 h after drug administration.

### Histopathology

All formalin-fixed, paraffin-embedded tissue samples were processed for hematoxylin and eosin (H & E) staining. Tumor diagnosis was established by an experienced neuropathologist according to the current World Health Organization (WHO) histopathological criteria (27). In our study, cell density and proliferation rate (Ki-67 labeling index) was investigated in each of the collected tissue samples.

### Preoperative Intake of AEDs

Since the main focus of our study was to investigate the potential influence of AEDs on 5-ALA induced fluorescence, we documented in each patient if AEDs were administered prior to surgery (yes or no). Patients were ingested average AED dosages: valproic acid (up to 1.5 mg per day), levetiracetam (800 mg per day). We did not study each drug separately, as the general series was not large.

### Postoperative Course

The neurological status of each patient was investigated before and after surgery to detect potential postoperative deterioration of neurological symptoms. Additionally, the extent of resection was judged according to the results of an early (up-to 72 h post-surgery) post-operative MRI: (1) GTR was present if at least 90% of the tumor mass was removed, (2) subtotal resection if more than 50% of the tumor mass was resected, and (3) partial resection if <50% of the tumor was removed.

### Statistical Analysis

Data processing was performed using R software for statistical computing (version 3.4.4). The continuous variables were compared in groups using Mann–Whitney *U*-test. To analyze the relationship between the categorical variables, a Fisher exact test was applied. A logistic regression analysis was performed to adjust for confounders when testing the effect of AED on fluorescence. The results were considered statistically significant for *p* < 0.05.

## Results

### 5-ALA Induced Fluorescence and Histopathological Diagnosis

We observed visible fluorescence during surgery in 14 (52%) of 27 cases, whereas no fluorescence was detected in the remaining 13 cases (48%).

**Figure 1:**
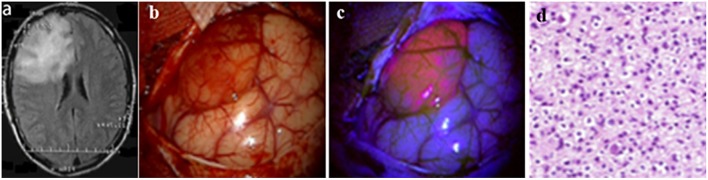
Illustrative case No. 16 (Diffuse fluorescence type). Oligodendroglioma WHO Grade II of the right frontal lobe. **(A)**–Preoperative FLAIR images show a large hyperintense lesion, **(B)**–White light microscopy shows distint cortical abnormalities; **(C)**–The tumor reveals moderate fluorescence with violet-blue excitation light; **(D)**–Histology reveals tumor tissue of a oligodenroglioma WHO grade II. Informed consent has been obtained from the patient for the publication of data, including images.

According to the histopathological tumor diagnosis, all 4 pilocytic astrocytomas, all 2 gemistocytic astrocytomas, and the only desmoplastic infantile ganglioglioma showed visible fluorescence. Furthermore, visible fluorescence was found in 4 (29%) of 14 diffuse astrocytomas, and 3 (50%) of 6 oligodendrogliomas. According to the intratumoral fluorescence homogeneity (*n* = 14 cases), 7 tumors had “diffuse” visible fluorescence, while the other 7 gliomas had “focal” sites of visible fluorescence. Details on the fluorescence data of our cohort are provided in Table 1 and illustrative cases in Figure 1.

**Table 1:** Intraoperative fluorescence characteristics.

**Histopathology**	**Fluorescence**					
	**Visible fluorescence**					**Absence of fluorescene**
	**Fluorescence grade**			**Fluorescence homogeneity**		
	**1-Mild**	**2-Moderate**	**3-Bright**	**Diffuse**	**Focal**	
Diffuse astrocytoma	1 (3.7%)	2 (7.4%)	1 (3.7%)	2 (7.4%)	2 (7.4%)	10 (37%)
Oligodendroglioma	1 (3.7%)	1 (3.7%)	1 (3.7%)	1 (3.7%)	2 (7.4%)	3 (11.1%)
Gemistocytic atrocytoma	–	–	2 (7.4%)	2 (7.4%)	0	0
Pilocytic astrocytoma	2 (7.4%)	1 (3.7%)	1 (3.7%)	1 (3.7%)	3 (11.1%)	0
Desmoplastic infantile ganglioglioma	–	1 (3.7%)	–	1 (3.7%)	0	0
In total:	4 (14.8%)	5 (18.5%)	5 (18.5%)	7 (25.9%)	7 (25.9%)	13 (48.1%)
In total:	14 (52%)			14 (51.8%)		13 (48%)
In total:	27 (100%)					

### 5-ALA Induced Fluorescence and Histological Parameters

Altogether, 80 tissue samples were collected during surgery from the 27 patients (median: 3 samples; range 1–12 specimens per patient). Of these, visible fluorescence was found in 21 samples (26%), whereas 59 samples (74%) showed no visible fluorescence. Cell density was significantly higher in fluorescence-positive samples (2,180 mm^2^) than in fluorescence-negative samples (1,510 mm^2^; *p* = 0.03). Additionally, the proliferation rate assessed by Ki-67 labeling index was also significantly higher in fluorescence-positive samples (2.52%) than in fluorescence-negative samples (0.41%; *p* = 0.04).

### 5-ALA Induced Fluorescence and AEDs

Due to a prior history of preoperative seizures, 15 patients (56%) of our study cohort were taking AEDs before surgery (finlepsin, levetiracetam, trileptal, and/or valproid acid). Of the 15 patients with preoperative AED intake, 11 patients (73%) showed no visible fluorescence during the resection. In contrast, 10 (83%) of the remaining 12 patients without prior AEDs intake showed visible fluorescence. Thus, visible fluorescence was significantly more common in patients without AEDs intake as compared to patients with preoperative AED intake [OR = 0.15 (CI 95% 0.012–1.07), *p* = 0.046]. The AED showed to be a significant factor (*p* = 0,048) influencing the probability of intraoperative fluorescence when adjusted for other potential confounders (astrocytoma/non-astrocytoma, frontal localization, dexamethasone dose) in a logistic regression model. The other factors included in the model did not show a significant relationship with the fluorescence (*p* > 0.05).

### Postoperative Course

After surgery, 21 patients showed stable and four patients improved neurological symptoms. In contrast, deterioration of neurological symptoms was found in the remaining two patients. According to early postoperative MRI, gross total resection was achieved in 16 patients (59%), subtotal resection in 8 patients (30%), and partial resection in three patients (11%). Fluorescence did not correlate with extent of resection.

## Discussion

The use 5-ALA induced fluorescence is becoming increasingly popular with the aim of maximizing the extent of resection especially in HGGs and thus improving postoperative patient prognosis. In this sense, fluorescence-guided surgery demonstrated to significantly improve the rate of complete resections in HGGs as compared to microsurgery alone, and this innovative technique also almost doubled the 6-months progression free-survival (16). Recently, visible PpIX fluorescence was found also in other brain lesions such as meningiomas (19), and metastatic tumors (18, 28).

### Current Literature: 5-ALA Fluorescence in LGG

So far, the value of 5-ALA induced fluorescence in LGG was only investigated in few studies. In this sense, Ishihara et al. first described in 2007 the application of 5-ALA in two LGG and did not find any visible fluorescence in the multiple collected tissue samples (29). Additionally, Ruge et al. published in 2009 a case report of a 9-year-old girl who underwent fluorescence-guided resection of a pleomorphic xanthoastrocytoma of the right temporal lobe and detected visible fluorescence of the tumor (20). Moreover, Stockhammer et al. published in 2009 a case report of a diffuse astrocytoma WHO grade II with moderate cellular density, higher microvascular density, and visible fluorescence (30). Cases of fluorescence of pilomyxoid astrocytoma and pilocytic astrocytoma were published by Bernal Garcia et al. (31) and Choo et al. (32), respectively. The first series of patients with LGG and 5-ALA was published by Widhalm et al. (33). In this study, all eight diffusely infiltrating WHO grade II gliomas did not show any visible 5-ALA induced fluorescence during surgery. In a further study, Ewelt et al. reported in 2011 visible fluorescence in only one (8%) of 13 LGGs during surgery (7). Three years after the first patient series, Widhalm et al. published a larger series in 2013 and found visible fluorescence in 4 (9%) of 33 LGGs (34). In another study by Marbacher et al., 8 (40%) of 20 LGGs showed visible fluorescence (18). In 2015, Valdes founded visible fluorescence in 4 (33%) of 12 analyzed LGGs (35). Finally, Jaber et al. found in 2016 in the largest series to date visible fluorescence in 13 (16%) of 82 LGGs (36). Thus, according to the current literature visible fluorescence is observed only in a minority of patients with LGGs.

### Current Study: 5-ALA Fluorescence in LGG

Despite of these other studies available in the current literature, our data showed the presence of visible fluorescence in more than half of our cases during surgery. One possible explanation for the higher rate of LGGs with visible fluorescence compared to the current literature might be that we used a slightly higher dose of 5-ALA (25 mg/kg bodyweight) as compared to the other studies (18, 33, 34). However, Stummer et al. compared different doses of 5-ALA in malignant glioma surgery (0.2, 2, 20 mg/kg) and concluded that usage of 5-ALA doses more than 20 mg/kg bodyweight would probably not result in an improved fluorescence effect (37). Another explanation might be that we included also other tumor entities apart from diffusely infiltrating gliomas such as pilocytic astrocytomas or one ganglioglioma. Our promising findings have to be confirmed, however, in independent multicenter studies including a larger cohort of patients suffering from LGG.

Interestingly, we did observe visible fluorescence not only in focal intratumoral areas as previously described (33, 34), but also diffuse fluorescence with homogeneous glowing of the whole tumor. We assume that presence of focal visible fluorescence in LGG might represent areas of potential future malignant transformation. Furthermore, future studies should clarify if the extent of resection could be optimized especially in LGGs with a diffuse fluorescence pattern.

### 5-ALA Fluorescence and Histopathology

In our study, we observed significantly higher levels of cell density and proliferation in samples with visible fluorescence as compared to no fluorescence. This is in line with the two previous studies by Widhalm et al (33, 34). Similarly, Widhalm et al found a significantly higher mitotic rate, cell density, and nuclear pleomorphism in fluorescing samples as compared to non-fluorescing specimens. Furthermore, the proliferation index assessed by MIB-1 LI was significantly higher in samples with visible fluorescence as compared to non-fluorescing specimens. In this sense, these previous data indicate that visible fluorescence is capable to identify anaplastic foci according to the WHO histopathological criteria (33, 34). Since we also found a significantly higher cell density and proliferation rate in areas of visible fluorescence in a series of LGG, we believe that 5-ALA might serve as an early marker of ongoing malignant transformation of an initial LGG. Future studies with sufficient data on follow-up are needed to clarify this important issue.

### 5-ALA Fluorescence and AEDs

Nowadays, it is common practice for patients with LGG to have medical treatment of epileptic seizures (38–40). Hefti et al. demonstrated in an *in-vitro* study that the PpIX synthesis was reduced by up to 45% in glioma cells under the effect of phenytoin, but not levetiracetam (26). In our study, we found that visible fluorescence was significantly more common in patients without AEDs intake as compared to patients with preoperative AED intake (*r* = 0.56; *p* = 0.045). Of the 15 patients with preoperative AED intake, 11 patients (73%) showed no visible fluorescence during the resection. In contrast, 10 (83%) of the remaining 12 patients without prior AEDs intake showed visible fluorescence. The underlying mechanisms for the observed influence of AEDs on visible fluorescence is unclear so far. The influence of AEDs on activities of 5-aminolevulinic acid dehydrase and uroporphyrinogen I synthetase in erythrocytes of a Vitamin B6-deficient epileptic boy given valproic acid and carbamazepine was described also by Haust et al. from University of Western Ontario, Canada in 1989 (41). One possible hypothesis might be that AEDs have an influence on the enzyms of the PpIX synthesis in the mitochondria of glioma cells and thus result in presence or absence of fluorescence in LGG. A further study on glioblastoma cell lines found that AEDs (phenytoin/valproates) and dexamethasone may inhibit PpIX synthesis (10). The exact mechanisms for the influence of AEDs on visible fluorescence have to be clarified in future studies.

### Future Directions

In future, our first observations should be confirmed in further independent studies with a large cohort of patients. Such larger studies allow also the possibility to analyze the influence of different types of AED separately. Furthermore, the influence of AEDs should also be investigated in HGGs. In cases with lack of visual fluorescence, quantitative detection methods might be useful for improved visualization of LGG tissue. In this sense, confocal microscopy is also a powerful tool to visualize cellular 5-ALA-induced tumor fluorescence within LGGs and at the brain-tumor interface (21). However, convincing data of confocal microscopy in a large cohort of patients with LGG are still missing so far. Another method represents the spectroscopic analysis of PpIX accumulation with specific probes. By this approach, Valdes et al. found that accumulation of PpIX can be detected quantitatively despite the poor diagnostic accuracy of visual fluorescence in LGGs (35, 42). Consequently, this promising approach warrants further investigation in future studies.

## Conclusions

In the present study, we investigated the role of 5-ALA in LGGs and the influence of antiepileptic drugs on intraoperative fluorescence. According to our data, we observed a markedly higher rate of visible fluorescence in our series of LGGs (52%) compared to current literature. Furthermore, increased cell density and proliferation was noted in areas of visible fluorescence. Thus, 5-ALA induced fluorescence might also improve intraoperative visualization of a subgroup of LGGs and might be also a useful marker for optimized detection of histopathological heterogeneity during surgery of LGGs. Furthermore, preoperative intake of AEDs seems to reduce the presence of visible fluorescence in such tumors according to our preliminary data and thus this issue should be taken into account in the clinical setting. Further, independent multicenter studies including a larger cohort of LGG patients are required to confirm the promising data of this present study.

## Limitations

We understand that the analysis of factors influencing Fluorescence should be multifold. To reach that, we used the model of logistic regression. AED proved to be a significant factor influencing the probability of intraoperative fluorescence, when adjusted for other potential confounders (astrocytoma/non-astrocytoma, frontal localization, dexamethasone dose, IDH1-mutation) in a logistic regression model. The cell density and Ki67 index were revealed only in patients with multiple biopsies (10 patients). It was concluded, that cell density, and Ki67 index were higher for the biopsy samples taken from the fluorescing zone compared to the non-fluorescing one. We've got little evidence on cell density for the rest 17 patients, with their Ki67 index making up mainly 4–5%. As this is a retrospective analysis enrolling 27 patients, we did not plan to study cell density and Ki67 index. Unfortunately, we've got no complete dataset for Ki67 and cell density to include into a logistic regression model.

## Ethics Statement

This study was carried out local committee of the N. N. Burdenko National Medical Research Center of Neurosurgery (Moscow, Russia) with written informed consent from all subjects. All subjects gave written informed consent in accordance with the Declaration of Helsinki. The protocol was approved by the local committee of the N. N. Burdenko National Medical Research Center of Neurosurgery (Moscow, Russia).

## Author Contributions

SG, GW, and AP designed the study. LS, VJ, and MR collected the date. TS, VL, GP, and AR worked out the technical details. MG and DC analyzed the data and wrote the paper with input from all authors. AS and AP edited the text. KC analyzed the data and wrote the paper with input from all authors.

### Conflict of Interest Statement

The authors declare that the research was conducted in the absence of any commercial or financial relationships that could be construed as a potential conflict of interest.
